# Tecovirimat Resistance in Mpox Patients, United States, 2022–2023

**DOI:** 10.3201/eid2912.231146

**Published:** 2023-12

**Authors:** Todd G. Smith, Crystal M. Gigante, Nhien T. Wynn, Audrey Matheny, Whitni Davidson, Yong Yang, Rene Edgar Condori, Kyle O’Connell, Lynsey Kovar, Tracie L. Williams, Yon C. Yu, Brett W. Petersen, Nicolle Baird, David Lowe, Yu Li, Panayampalli S. Satheshkumar, Christina L. Hutson

**Affiliations:** Centers for Disease Control and Prevention, Atlanta, Georgia, USA (T.G. Smith, C.M. Gigante, N.T. Wynn, A. Matheny, W. Davidson, Y. Yang, R.E. Condori, K. O’Connell, L. Kovar, T.L. Williams, Y.C. Yu, B.W. Petersen, N. Baird, D. Lowe, Y. Li, P.S. Satheshkumar, C.L. Hutson);; Deloitte Consulting LLC, Arlington, Virginia, USA (K. O’Connell); Leidos Inc., Reston, Virginia, USA (L. Kovar)

**Keywords:** mpox, viruses, antivirals, tecovirimat, ST-246, TPOXX, monkeypox virus, orthopoxvirus, antimicrobial resistance, United States

## Abstract

During the 2022 multinational outbreak of monkeypox virus (MPXV) infection, the antiviral drug tecovirimat (TPOXX; SIGA Technologies, Inc., https://www.siga.com) was deployed in the United States on a large scale for the first time. The MPXV F13L gene homologue encodes the target of tecovirimat, and single amino acid changes in F13 are known to cause resistance to tecovirimat. Genomic sequencing identified 11 mutations previously reported to cause resistance, along with 13 novel mutations. Resistant phenotype was determined using a viral cytopathic effect assay. We tested 124 isolates from 68 patients; 96 isolates from 46 patients were found to have a resistant phenotype. Most resistant isolates were associated with severely immunocompromised mpox patients on multiple courses of tecovirimat treatment, whereas most isolates identified by routine surveillance of patients not treated with tecovirimat remained sensitive. The frequency of resistant viruses remains relatively low (<1%) compared with the total number of patients treated with tecovirimat.

In May 2022, an outbreak of mpox disease, caused by infection with monkeypox virus (MPXV) clade IIb (formerly West Africa clade), was identified in the United States (*1*). Since that time, >30,000 cases and 46 deaths associated with the outbreak have been identified in the United States. As a result of effective education, vaccination, and case identification, US cases peaked the first week of August 2022 at 459 cases per week. The United States has identified more cases than any other country in the global outbreak (*2*).

The FDA licensed the therapeutic agent TPOXX (SIGA Technologies, Inc., https://www.siga.com) containing the drug tecovirimat (i.e., ST-246) under the animal rule for smallpox treatment in 2018 (*3*). Tecovirimat has been tested extensively in cell culture (*4*–*6*) and within many orthopoxvirus (OPXV) animal models (*7*–*15*), including the nonhuman primate variola virus (VARV) model (*16*,*17*). After MPXV clade IIb emerged in 2022, Warner et al. demonstrated tecovirimat efficacy against the outbreak strain (lineage B.1) in a nonlethal mouse model (*18*). Although tecovirimat has shown efficacy against multiple OPXVs, researchers have noted that nucleotide alterations to the orthopoxviral F13L gene homologue leading to amino acid substitutions in the F13 protein (also known as VP37) allow for resistance (*4*,*19*). In addition, resistance emerged during use of tecovirimat in an extended treatment course of a patient with progressive vaccinia (*20*).

Because TPOXX is only licensed for treatment of smallpox, the Centers for Disease Control and Prevention (CDC) holds an expanded access investigational new drug protocol for treatment of nonvariola OPXV infections, including mpox. Since May 2022, at least 7,563 patients have received tecovirimat for mpox treatment in the United States; a fraction of those have been severe cases where patients have moderate to severe immunocompromise usually caused by uncontrolled HIV infection (*21*). To test for resistance among patients who received tecovirimat, we collected specimens from 435 patients who received tecovirimat for whom resistance was possible or suspected based on clinical data (Table 1). We genotyped and phenotyped specimens from 68 patients and confirmed a resistant phenotype in 46 of those patients. The geographic distribution of tecovirimat resistance has conformed to the geographic distribution of the larger mpox outbreak (Figure 1). Here, we describe our investigation and findings. The activities in this report were reviewed by the Human Subjects Advisor in the National Center for Emerging and Zoonotic Diseases at the Centers for Disease Control and Prevention and determined that it does not meet the regulatory definition of research under provision 45 CFR 46.102(l); the activities fall under public health surveillance and do not require IRB review. 

**Table 1:** Surveillance for tecovirimat resistance in mpox cases, United States, 2023*

Category	No. samples or isolates	No. patients
Genomic testing		
Sequences analyzed	3,247	
F13 substitutions other than E353K found (Table 2)	130	76
Phenotype testing		
Submitted to CDC	801	435
MPXV isolated	164	83
Phenotype testing complete	124	68
Tecovirimat resistant	96	46
HIV-positive		39
CD4+ T-cell count <350 cell/µL		31
CD4+ T-cell count <200 cell/µL		28
Deceased		10
Hospitalized		34
Tecovirimat treated		39

*CDC, Centers for Disease Control and Prevention; MPXV, monkeypox virus.

**Figure 1:**
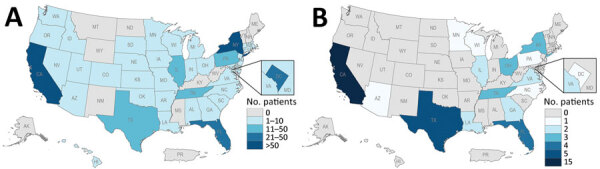
Geographic distribution of patients with mpox who had samples received for tecovirimat resistance testing (A) and who had samples confirmed resistant (B) June 2022–July 2023, United States.

## Methods

During the mpox outbreak, whole-genome metagenomic sequencing and, more recently, amplicon-based sequencing targeting the F13L gene have been used to screen for changes in the MPXV F13L homologue (Appendix 1). A total of 3,247 CDC-generated sequences have been screened by either passive genomic surveillance (n = 3,101) or targeted F13L sequencing (n = 146). Only genomic sequencing completed at CDC was included because the raw data were required to find minor variants. The primary outbreak strain (MPXV clade IIb lineage B.1) has a substitution, E353K, in the F13 protein that is not present in the secondary outbreak strain (lineage A.2), historical clade IIb sequences from Nigeria, or MPXV clade IIa (*22*). Because the E353K substitution was not previously described in other OPXV, the effect on tecovirimat phenotype was unknown. 

We adapted a cytopathic effect (CPE) assay, used at CDC to test VARV sensitivity to tecovirimat, to use for MPXV as described previously (*22*). In brief, we used clinical specimens that were decoded but not anonymous to culture MPXV on Vero (African green monkey) cell lines (either BSC-40 or E6). We then used the isolated MPXV to infect confluent Vero E6 cell monolayers pretreated for 1 h with different concentrations of tecovirimat. We incubated infected cells for 72 h at 35°C with 6% CO_2_. We fixed and stained wells with formalinized crystal violet and measured absorbance at 570 nm; intact cell monolayers having a high absorbance indicated that the drug was protective. We used the CPE assay to show that MPXV isolates with the E353K mutation remained sensitive to tecovirimat (*18*,*22*–*24*).

## Results

In total, 130 samples from 76 patients produced sequences with amino acid changes other than E353K in the F13 protein relative to MPXV clade IIb variant B.1 reference strain (GenBank accession no. ON563414), collected in the United States in 2022 (Table 2). Isolates with amino acid substitutions D100N, D217N, D248N, D256N, and S369L identified by routine sequencing of samples from patients not treated with tecovirimat have remained sensitive (Table 2). We confirmed 11 amino acid mutations (H238Q, Y258C, N267D, N267del, D283G, A288P, A290V, D294V, A295E, L297ins, I372N) that were previously identified in other OPXV (*19*,*20*,*25*–*28*) as resistant by phenotypic testing (Table 2). One confirmed resistance mutation, T289A, had not been described before the 2022 mpox outbreak (*26*). T289A resulted in up to an 8-fold increase in the 50% effective concentration when compared with the MPXV clade IIa reference strain. This position is part of the predicted tecovirimat binding site and adjacent to A288P and A290V, which both confer resistance (*19*). We identified 7 other amino acid substitutions (K174N, S215F, P243S, T245I, Y285H, R291K, D301del) but have not yet determined the effects of those mutations (*25*,*26*). Those mutations have been observed and tested only in combination with other resistance mutations. 

**Table 2:** MPXV F13 mutations identified from 76 patients with mpox, United States, 2023*

Amino acid substitution	Isolates	Patients	EC_50_, µmol/L	Fold change†
A288P (*25**,**26*)	6	4	0.5 to >500	29 to >29,000
A288P, A290V, D294V (*26*)	3	1	0.66 to >500	38 to >29,000
A288P, A290V, L297ins (*26*)	1	1	>500	>29,000
A288P, A290V, I372N	1	1	15	880
A288P, D294V, A295E	1	1	1.4	83
A288P, D294V, D301del (*26*)	1	1	>500	>29,000
A288P, I372N	1	1	>150	>8,600
A290V (*25*,*26*)	9	9	0.17–43	10–2,500
A290V, I372N (*25*)	5	5	30–32	1,700–1,800
A295E	3	3	2.0–3.3	110–190
D100N	2	2	0.008	−2
D217N	11	11	0.007–0.012	−2.4 to −1.3
D248N	1	1	0.007	−2.4
D256N	3	3	0.009	−1.8
D283G	2	1	7.1–7.3	404–420
D294V (*25*)	8	7	0.23–1.4	13–78
D294V, A295E	1	1	1	59
H238Q (*25*)	4	4	0.54–0.6	28–34
H238Q, A288P, D294V, I372N (*25*)	1	1	≈5.2	≈290
H238Q, N267D, A295E	1	1	24	1,400
I372N (*25*)	12	9	0.04–>150	2.3 to >8600
K174N, N267D	1	1	12	720
N267D (*25*)	3	3	10–11	570–630
N267D, A288P (*25*,*26*)	4	3	1.2–16	71–900
N267D, A290V	1	1	2.0	110
N267D, D294V	1	1	12	680
N267D, A288P, A290V, D294V (*26*)	1	1	>500	>29,000
N267D, A288P, A290V, A295E, L297ins (*26*)	1	1	>500	>29,000
N267D, A288P, A290V, A295E, I372N	1	1	>500	>29,000
N267del (*27*)	8	7	1.5–4.0	85–230
N267del, N267D	1	1	Not tested	
N267del, N267D, A295E	2	2	2.9–18	160–1,000
N267del, N267D, A288P, A295E	1	1	Not tested	
N267del, N267D, D294V, A295E	1	1	2.5	140
N267del, A288P, A295E	1	1	>500	>29,000
N267del, T289A, A295E	1	1	0.26	15
N267del, A290V	1	1	0.13	7.5
N267del, A290V, I372N	1	1	3.1	180
P243S, A288P, A290V (*26*)	1	1	0.56	32
S215F, T289A, A290V, I372N	1	1	Not tested	
S369L	3	3	0.006	−2.9
T245I, A290V	1	1	0.17	10
T289A	3	3	0.078–0.14	3.7–7.8
T289A, I372N	1	1	Not tested	
T289A, R291K	1	1	1.7	98
Y258C	1	1	18	1,000
Y285H, I372N	1	1	0.045	2.6

*All specimens belong to MPXV clade IIb lineage B.1 and contain E353K substitution in addition to the listed substitutions. All substitutions detected from a specimen are listed regardless of their proportion in the viral population. Insertions (ins) and deletions (del) were detected in addition to substitutions. EC_50_, 50% effective concentration; MPXV, monkeypox virus. †Fold change was calculated based on the EC_50_ of the reference strain MPXV clade IIa (U.S., 2003), which was 0.0175 µmol/L.

Eight of 27 nonsynonymous mutations observed in F13L were GA to AA or TC to TT, which may suggest they arose through APOBEC3 editing. All the APOBEC3 motif mutations produced amino acid changes that did not affect tecovirimat resistance in culture. Resistance phenotype is currently unknown for R291K, S215F, and P243S.

For phenotype testing, we considered an isolate resistant if the increase in 50% effective concentration was ≥2-fold compared to the 2003 MPXV clade IIa reference strain. Isolates with 2-fold to 9-fold change were considered partially resistant, and isolates with >10-fold change were considered resistant (*25*). A total of 83 isolates from 41 patients were resistant, and 16 isolates from 11 patients were partially resistant. Four patients with partially resistant isolates also had >1 other isolate that was resistant. The clinical relevance of partially resistant and resistant isolates remains unknown.

## Discussion

**Figure 2:**
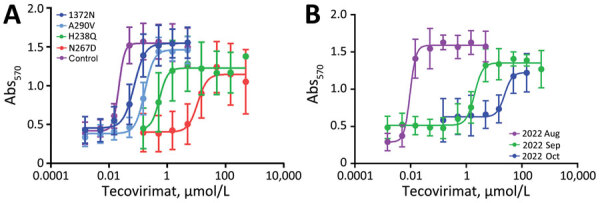
Examples of tecovirimat resistance in mpox patients, United States, 2022–2023. Patient samples were sequenced, cultured, and subjected to tecovirimat sensitivity testing in a cytopathic effect assay. A) Different samples from the same patient showed different F13 amino acid substitutions that result in different levels of resistance compared with the wild-type control (MPXV clade IIa, collected in the United States in 2003 [GenBank accession no. ON563414]). B) Samples from the same patient at different times before and after starting tecovirimat treatment in August 2022, showing sensitivity before drug treatment and increasing resistance after drug treatment. Abs_570_, absorbance at 570 nm.

Multiple lines of evidence point to tecovirimat resistance developing during drug treatment in most patients. First, genome sequencing has revealed unique mutational profiles from different sample sites from the same patient (Figure 2, panel A), indicating different viral subpopulations were selected at different sites during treatment. Second, longitudinal sampling was investigated for 4 of the 46 patients with a resistant isolate and showed samples before tecovirimat treatment were sensitive, whereas later samples were resistant (Figure 2, panel B). An exception was found for 1 patient; T289A was detected in 58% of reads, along with minor populations of A295E (9%) and N267del (22%), from a sample the day before the patient started tecovirimat treatment. A second sample from the same patient after tecovirimat treatment showed the T289A mutation was selected (93%), and a new variant R291K was also detected (31%). In addition, N267del was detected in a cluster of cases in California with no known tecovirimat treatment (*27*). Whether those drug-resistant infections were acquired from another person treated with tecovirimat is unknown but is a viable hypothesis. Such rare cases show that viruses with mutations in F13L resulting in tecovirimat resistance can be transmitted from person to person.

For patients that had >1 specimen with confirmed tecovirimat resistance, 39/46 had HIV infection; HIV status was unknown for the remaining 7 patients. Of the 39 patients with HIV infection, 31 had a CD4+ T-cell count available; all 31 were <350 cells/µL, and 28 were <200 cells/µL. Ten of the 39 patients died (*25*,*26*), and all 10 deceased patients had CD4+ T-cell counts <200 cell/µL. In the United States, 46 deaths associated with mpox have been reported (*2*). In those patients, severe immunocompromise is resulting in severe disease and death as well as tecovirimat resistance.

For patients who had >1 specimen with confirmed tecovirimat resistance, at least 34 of 46 patients were hospitalized. No medical history was available for 6 patients, and the medical history concerning hospitalization was not clear or did not mention hospitalization for another 6 patients. Of the 46 patients, 39 patients received tecovirimat either oral or IV; 5 patients did not receive tecovirimat (*27*), and 2 patients’ tecovirimat status was unknown. Exact data on length of tecovirimat exposure is difficult to obtain because of possible noncompliance with oral administration and multiple rounds of treatment in which drug administration stops and starts. We estimated the average length of tecovirimat treatment using the reported start date of tecovirimat treatments. Dates were available for 28 of the 39 patients that received tecovirimat (Appendix Table). The average length of tecovirimat treatment was 39 days (range 14–167 days); a standard regimen is 14 days. 

A tecovirimat-resistant phenotype was previously published for 6 patients from Los Angeles County, California, USA (*25*,*26*). The previous case reports were limited in geographic scope, whereas our study is an overview for the entire United States. The 6 patients previously reported are included in this report for completeness. The larger dataset reported herein is complementary to the previously published data and supports the conclusions of the previous reports. In addition, 1 other case report found MPXV with a tecovirimat-resistant phenotype that was linked to selection of the N267D mutation during tecovirimat treatment (*29*). Of note, despite detection of drug resistance from 1 anatomic site, the patient improved clinically (*29*). Other case reports have suspected tecovirimat resistance on the basis of deteriorating clinical status after tecovirimat treatment (*30*,*31*). Treatment with cidofovir was successful in those cases and should be considered when tecovirimat resistance is suspected.

The first limitation of our study is that the phenotype assay is culture-based, which is labor intensive and of low throughput. As of July 2023, we had phenotyped 124 specimens from 68 patients. However, the lag in testing means all the specimens that have been tested are from September 2022–April 2023, so results only give a retrospective sample of possible drug resistance. Because submission of samples for tecovirimat sensitivity is voluntary and cannot be used to inform clinical care, sampling bias may exist for certain physicians, hospitals, or states and may make it appear that certain states have more drug resistance than others (Figure 1). As genomic sequencing has increased, we have prioritized samples with predicted resistance mutations for phenotype testing. Mixed populations of cultured virus were tested to meet the need for efficiency for a public health emergency. In the future, we will begin plaque purification for selected samples to test clonal populations.

Our results confirm that tecovirimat resistance mutations are being selected in human mpox patients by tecovirimat treatment. Resistance has been confirmed in a small percentage of cases, currently <1% of the total number of patients that have received tecovirimat. Characteristics of patients with resistant isolates are very similar: uncontrolled HIV infection with very low CD4+ T-cell counts and potential for extensive tecovirimat exposure while hospitalized. The frequency of tecovirimat resistance may be higher in persons with uncontrolled HIV infection. In rare cases, a drug-resistant virus appeared to have been transmitted to another person. Genomic and phenotype testing are ongoing. Our results may be useful when considering treatment for patients that match the clinical profile we described; aggressive early dosing and combination therapy regimens could be considered in those instances (*21*). Results will also provide critical knowledge to potentially build a genomic assay for early detection of resistance mutations which could be used to inform clinical care decisions. For clinicians concerned about tecovirimat resistance, we encourage enrolling patients in the CDC VIRISMAP study (https://www.cdc.gov/poxvirus/mpox/clinicians/virismap.html) and the STOMP (Study of Tecovirimat for Mpox) trial (https://stomptpoxx.org).

In conclusion, we describe a large number of tecovirimat-resistant MPXV isolates from humans and provide crucial data on the amino acid changes leading to resistance in MPXV paired with clinical outcomes; these combined data may inform decisions on tecovirimat use in the future. Our findings also highlight the need for additional, well-tolerated OPXV therapeutics with different modes of action, particularly for use with immunocompromised patients. 

Appendix Additional information about tecovirimat resistance in mpox patients, United States, 2022–2023.
