# Supplementary material for: Tecovirimat Resistance in Mpox Patients, United States, 2022–2023
Source: Emerg Infect Dis. 2023 Dec;29(12):2426–32. doi: 10.3201/eid2912.231146 (PMC10683829; doi:10.3201/eid2912.231146)
Supplement: Appendix — Additional information about tecovirimat resistance in mpox patients, United States, 2022–2023. [file 23-1146-Techapp-s1.pdf]

Article DOI: http://doi.org/10.3201/eid2912.231146

EID cannot ensure accessibility for supplementary materials supplied by authors. Readers who have difficulty accessing supplementary content should contact the authors for assistance.

Tecovirimat Resistance in Mpox Patients, United States, 2022–2023

Appendix

Additional Methods

DNA was extracted from lesion swabs using EZ-1 DNA tissue kit (Qiagen) followed by heat inactivation at 56°C for ≥1 hour. Monkeypox virus infection was confirmed by real-time PCR using a clade II-specific monkeypox virus real-time PCR assay as described in Li et al. (*1*).

F13L Amplicon Sequencing

5 µL of MPXV DNA was used as input to the primary PCR reaction with tagged primers (F13L Forward: ont_tag-GACCTTCTTCATTTCGTGCCA, F13L: Reverse ont_tag-AATGTGGCCATTTGCATCGG), where ont_tag was added as described by the manufacturer (Oxford Nanopore Technologies, EXP-PBC096). Reaction contained 10 µL 2x GC Buffer I (Takara, RR02AG), 0.2 µL long amplicon Taq polymerase (Takara, RR02AG), 0.5 µL each of F13L forward and reverse primers at 20 µM, 2 µL dNTPs (Takara, RR02AG), and 1.8 µL nuclease-free water. PCR reaction was run for 2 minutes at 94°C followed by 25 to 35 cycles of 30 s at 94°C, 30 s at 56°C, and 1 minute at 72°C, followed by 5 minutes at 72°C. Cycle number was determined by Clade II-specific Ct value: 25 cycles for Ct 20 – 25, 30 times for Ct 25 – 30, and 35 cycles for Ct >30. Samples with Ct <20 were diluted 100-fold then run for 25 cycles. PCR reactions were cleaned up with 0.65x AMPure XP beads (Beckman). Barcoding PCR was performed according to the manufacturer's instructions (Oxford Nanopore Technologies, EXP-PBC096) using 20 µL reactions, Takara LA taq with GC buffers as above (Takara, RR02AG), 1 minute extension time and 12 cycles of PCR. PCR reactions were cleaned up with 0.65x AMPure XP beads (Beckman). Samples concentrations were estimated using a Qubit and pooled at equal concentrations. Library preparation was performed using the SQK-LSK109 kit for sequencing on the Flongle device, according to the manufacturer (Oxford Nanopore

Technologies). Basecalling was performed using guppy 6.1.2 (Oxford Nanopore Technologies) and flags –kit SQK-LSK109 –flowcell FLO-FLG001 –barcode_kits EXP-PBC096 –trim_barcodes –require_barcodes_both_ends. Nanopore reads were trimmed to remove 55 bp from each end (seqtk 1.0, https://github.com/lh3/seqtk) and all reads below 50 bp were removed (trimmomatic 0.39, https://github.com/timflutre/trimmomatic) before mapping to MPXV Nigeria reference MT903344 with 6,000 bp removed from the left terminus using minimap2 2.16 (https://github.com/lh3/minimap2). Variants were called using ivar 1.3.1 (https://andersen-lab.github.io/ivar/html/manualpage.htmlhid) and samtools 1.7 (https://github.com/samtools/samtools) using the command samtools mpileup -aa -A -B -Q 0 -d 80000 mappingfile.bam | ivar variants -p samplename -t 0.05 -m 5 -q 20.

Illumina Metagenomics Sequencing

Extracted DNA (15 µL) was used as input for the Illumina DNA Prep method according to the manufacturer's protocol except one half reagent volumes were used throughout. Libraries were visualized using an Agilent Fragment Analyzer instrument and an HS NGS Fragment Kit (Agilent Technologies Inc., Santa Clara, CA). Forty-eight samples were pooled at approximately equal molarity generating 200 pM final loading concentration and sequenced on an Illumina NovaSeq 6000 instrument using the 300 cycle SP sequencing components. Orthopoxvirus reads were filtered using Kraken2 v2.1.2 (*2*) run with default settings, using a database that included human genome for negative selection and MPXV genomes for positive selection. We used seqtk v1.3 `subseq` (*3*) to subsample our reads to orthopoxvirus with default settings and the '—no-name' flag, then used fastp v0.23.2 (*4*) with to trim and clean our filtered reads. Reads were aligned to MPXV Clade IIb reference genome (UK-P2; MT903344.1) using bwa mem v0.7.17 (H. Li et al., unpub. data, https://doi.org/10.48550/arXiv.1303.3997) then sorted using Samtools v.1.15.1 (*5*). F13L variants were called using iVar v.1.3.1 (*6*) with the following parameters: samtools mpileup -aa -A -d 600000 -B -Q 0 $PREFIX.BAM | ivar variants -p $PREFIX -r $REFERENCE -q 20 -t 0.05 -m 5. We converted from tsv to vcf format using a custom python script (https://github.com/jts/ncov-tools/blob/master/workflow/scripts/ivar_variants_to_vcf.py), filtering for an allele frequency of 0.05 and >5 supporting reads. For both ONT and Illumina data, only variants with allele frequency >10% are reported here (Appendix Table).

References

1. Li Y, Zhao H, Wilkins K, Hughes C, Damon IK. Real-time PCR assays for the specific detection of monkeypox virus West African and Congo Basin strain DNA. J Virol Methods. 2010;169:223–7. PubMed https://doi.org/10.1016/j.jviromet.2010.07.012

2. Wood DE, Lu J, Langmead B. Improved metagenomic analysis with Kraken 2. Genome Biol. 2019;20:257. PubMed https://doi.org/10.1186/s13059-019-1891-0

3. Li H. Toolkit for processing sequences in FASTA/Q formats. GitHub. 2012 [cited 2023 Apr 12]. https://github.com/lh3/seqtk

4. Chen S, Zhou Y, Chen Y, Gu J. fastp: an ultra-fast all-in-one FASTQ preprocessor. Bioinformatics. 2018;34:i884–90. PubMed https://doi.org/10.1093/bioinformatics/bty560

5. Li H, Handsaker B, Wysoker A, Fennell T, Ruan J, Homer N, et al.; 1000 Genome Project Data Processing Subgroup. The Sequence Alignment/Map format and SAMtools. Bioinformatics. 2009;25:2078–9. PubMed https://doi.org/10.1093/bioinformatics/btp352

6. Castellano S, Cestari F, Faglioni G, Tenedini E, Marino M, Artuso L, et al. IVar, an interpretation-oriented tool to manage the update and revision of variant annotation and classification. Genes (Basel). 2021;12:384. PubMed https://doi.org/10.3390/genes12030384

7. Alarcón J, Kim M, Terashita D, Davar K, Garrigues JM, Guccione JP, et al. An Mpox-Related Death in the United States. N Engl J Med. 2023;388:1246–7. PubMed https://doi.org/10.1056/NEJMc2214921

8. Garrigues JM, Hemarajata P, Karan A, Shah NK, Alarcón J, Marutani AN, et al. Identification of Tecovirimat Resistance-Associated Mutations in Human Monkeypox Virus - Los Angeles County. Antimicrob Agents Chemother. 2023;67:e0056823. PubMed https://doi.org/10.1128/aac.00568-23

Appendix Table. F13 mutations identified in15 mpox cases, United States, 2022*

Patient	Specimen	Mutation	Illumina	ONT	Difference	Illumina depth	ONT depth	Days of treatment	Sample day
1	1	N267D	100.00%	98.90%	1.10%	37	366	28	51
2†	1	A288P	26.90%	29.30%	−2.40%				
		D294V	26.10%	18.00%	8.10%	2831	1617	28	31
		D301del	23.60%	17.10%	6.50%				
2†	2	A288P	18.59%	19.41%	−0.82%				
		A290V	26.88%	26.50%	0.38%	1926	4670	28	31
		D294V	24.80%	16.64%	8.16%				
2†	3	A288P	13.97%	14.78%	−0.81%				
		A290V	20.61%	22.44%	−1.83%	1446	5723	28	31
		L297ins	37.59%	33.22%	4.36%				
3	1	D294V	100.00%	96.60%	3.40%	75	70	36	38
4	1	T289A	100.00%	100.00%	0.00%	71	162	NA	NA
5	1	D294V	99.20%	98.20%	1.00%	118	148	19	56
6	1	N267del	22.20%	20.00%	2.20%				
		T289A	58.20%	61.00%	−2.80%	90	5962	0	0
		A295E	9.46%	11.20%	−1.74%				
6	2	T289A	92.90%	89.90%	3.00%	114	4461	18	18
		R291K	30.70%	34.10%	−3.40%				
7	1	N267D	59.11%	66.59%	−7.48%	291	317	75	91
		D294V	29.51%	17.02%	12.49%				
7	2	N267del	89.81%	72.85%	16.97%	373	1563	75	91
8	1	A295E	100.00%	98.52%	1.48%	37	2456	35	26
8	2	N267del	47.79%	30.68%	17.11%				
		A288P	22.06%	28.22%	−6.16%	66	138	35	26
		A295E	15.15%	27.11%	−11.96%				
9	1	N267D	25.42%	29.78%	−4.35%				
		A288P	ND	11.76%	missed				
		A290V	16.24%	12.00%	4.24%	118	131	28	65
		A295E	21.01%	11.61%	9.40%				
		I372N	17.53%	15.26%	2.26%				
10	1	A290V	95.00%	84.29%	10.71%	20	5377	77	87
		T245I	ND	11.75%	missed				
11‡	1	A290V	100.00%	97.80%	2.20%	161	3373	52	60
11‡	2	I372N	100.00%	85.72%	14.28%	334	5176	52	60
12	1	N267D	18.32%	28.07%	−9.75%	198	418	30	43
		A295E	60.32%	56.01%	4.31%				
12	2	N267del	78.40%	67.94%	10.46%	125	4824	30	43
13‡	1	A288P	54.94%	53.75%	1.19%	134	1241	28	32
		N267D	43.28%	48.72%	−5.44%				
14	1	Y285H	10.13%	8.83%	1.30%	60	4092	56	84
		I372N	90.00%	77.04%	12.96%				
15	1	D217N	100.00%	98.97%	1.03%	19	91	NI	NI

*DNA extracted from each specimen was sequenced by direct DNA sequencing on an Illumina NovaSeq 6000 or targeted F13L amplicon sequencing on an Oxford Nanopore MinIon. Percent of reads with each mutation is shown for the two methods. Two minor alleles were not detected by the direct DNA sequencing method (ND not detected). Allele frequencies less than 10% were not reported unless it was detected at >10% by the other method. Average read depth is included. For some cases, multiple specimens collected from different anatomic sites yielded different mutational patterns. Amino acid deletion (del) and insertion (ins) mutations are included. Length of treatment indicates the potential number of days of tecovirimat exposure. Sample day indicates the number of days from initial mpox diagnosis to resistant sample collection. One patient's medical history was not available (NA), and one was not investigated (NI) because the sample was sensitive to tecovirimat.

†Previously published by Alarcón, et al. (*7*).

‡Previously published by Garrigues, et al. (*8*).
